# P-747. Predictive Factors of Poor Outcomes in Non-Necrotizing Bacterial Dermohypodermitis: A Retrospective Study

**DOI:** 10.1093/ofid/ofaf695.958

**Published:** 2026-01-11

**Authors:** Amal Chakroun, Fatma Hammami, khaoula Rekik, Makram Koubaa, fatma smaoui, chakib marrakchi, Mounir Ben Jemaa

**Affiliations:** Infectious Diseases Department, sfax, Sfax, Tunisia; Infectious Diseases Department, Hedi Chaker University Hospital, University of Sfax, Tunisia, Sfax, Sfax, Tunisia; Infectious Diseases Department, sfax, Sfax, Tunisia; Infectious Diseases Department, Hedi Chaker University Hospital, University of Sfax, Tunisia, Sfax, Sfax, Tunisia; Infectious Diseases Department, sfax, Sfax, Tunisia; Infectious Diseases Department, sfax, Sfax, Tunisia; Infectious Diseases Department, Hedi Chaker University Hospital, University of Sfax, Tunisia, Sfax, Sfax, Tunisia

## Abstract

**Background:**

Non-necrotizing bacterial dermohypodermitis (NBDH) is a common cause of hospitalization, typically responding well to appropriate antibiotic therapy. However, in some cases, the course may be complicated by local or systemic infectious events, potentially affecting prognosis. We aimed to describe the clinical course and identify predictive factors of unfavorable outcomes in patients hospitalized with NBDH.

**Methods:**

We conducted a retrospective study including adult patients admitted for NBDH in the Infectious Diseases Department of Hedi Chaker University Hospital (Sfax, Tunisia) between January 2015 and December 2024. A favorable outcome was defined by defervescence within 72 hours and improvement or resolution of local signs within 5 days. An unfavorable outcome was defined as lack of clinical improvement after 5 days, or the occurrence of complications (e.g., sepsis, necrosis, abscess) or death

**Results:**

A total of 115 patients were included (mean age: 59 ± 16 years; 52.2% female). A cutaneous portal of entry was identified in 47% of cases. Empirical antibiotic therapy was mainly based on amoxicillin–clavulanate plus clindamycin (34.8%). Microbiologically guided adjustment was required in 8 cases. Surgical intervention was necessary in 5 cases. Mean antibiotic duration was 14 ± 7.2 days. Overall, 82.6% of patients had a favorable outcome. Independent predictors of unfavorable outcome included age >65 years (p=0.0056), obesity (p=0.008), and chronic venous insufficiency (p=0.052). Female sex (p=0.63), diabetes (p=0.523), and prior episodes of NBDH (p=0.413) were not significantly associated. Recurrence occurred in 9.9% of cases.

**Conclusion:**

Early identification of risk factors such as advanced age, obesity, and venous insufficiency is essential for optimizing NBDH management and improving patient outcomes

**Disclosures:**

All Authors: No reported disclosures

